# Analyses of exon 4a structure reveal the properties of Big tau related to distribution, function and aggregation

**DOI:** 10.3389/fnmol.2025.1707820

**Published:** 2025-10-28

**Authors:** Itzhak Fischer, Peter W. Baas

**Affiliations:** Department of Neurobiology and Anatomy, Drexel University College of Medicine, Philadelphia, PA, United States

**Keywords:** tau, microtubule-associated protein, exon 4a, hydrophobicity, β-sheet, protein aggregation, neurodegeneration, evolutionary conservation

## Abstract

Tau, a microtubule-associated protein that modulates the dynamic properties of microtubules, is best known for its involvement in tauopathies. Usually expressed as the low molecular (LMW) variants of 45–60 kDa, tau is also expressed as a high molecular weight isoform of 110 kDa, termed Big tau, in neurons of the peripheral nervous system and in a few types of central neurons. Big tau is defined by the inclusion of exon 4a, which adds about 250 amino acids to the projection domain. Despite low sequence conservation the length of the Big tau insert remains remarkably consistent across vertebrates. Here, we analyzed the charge distribution, hydrophobicity, and aggregation propensity of the human sequences of LMW tau, Big tau and the amino acids encoded by exon 4a. Exon 4a amino acids display a pronounced negative net charge of acidic amino acids, an overall hydrophilic composition and low β-sheet content. This contrasts with LMW tau, which is more hydrophobic with extended aggregation-prone motifs including a relatively high β-sheet content. Inclusion of exon 4a in Big tau shifts the global hydrophobicity to intermediate values and reduces predicted β-sheet content, suggesting decreased aggregation propensity. We propose a model in which inclusion of the additional stretch of amino acids encoded by exon 4a shields the aggregation motifs of LMW tau and limits their exposure, which together with its unique biophysical structure, defines the properties of Big tau, Evolutionary analyses across vertebrates (human, rat, zebra finch, frog) confirms the minimal sequence identity and conserved exon size but shows preservation of negative net charge indicating convergent retention of charge-based properties. Hydrophilicity was also broadly conserved, though less invariant across species. These results are consistent with the presence of Big tau in neurons that are resistant to tauopathies that commonly afflict neurons expressing only LMW tau.

## Introduction

Tau proteins are microtubule-associated proteins that regulate cytoskeletal dynamics, axonal transport, and synaptic function (Baas and Qiang, 2019; Alonso et al., 2024). Their pathological aggregation into abnormal filaments is a defining feature of several neurodegenerative diseases collectively termed tauopathies (Avila, 2006; Wang and Mandelkow, 2016). The aggregation process of tau is complex and includes not only aggregation-prone domains of the protein, but is also driven by mutations, posttranslational modifications, cellular stress factors and tau fibrils acting as seeds (Limorenko and Lashuel, 2021).

Among tau isoforms, Big tau is distinguished by the inclusion of exon 4a, an alternative exon encoding ∼250 amino acids (Goedert et al., 1992; Boyne et al., 1995; Fischer and Baas, 2020). The tau 4a exon distinguishes Big tau from its lower molecular weight (LMW) counterparts, but the resulting alterations in tau function have remained enigmatic since its discovery in the early 1990s. What is established is that the large size of the stretch of approximately 250 amino acids encoded by exon 4a (hereafter referred to as exon 4a protein) dramatically extends the projection domain and increases the overall size of the tau protein by nearly 60% compared to LMW tau. This major extension is likely to affect both the structural and functional properties of tau. Indeed, Big tau is selectively expressed in the peripheral and autonomic nervous systems, as well as in distinct regions of the central nervous system such as the cerebellum and brainstem (Boyne et al., 1995; Fischer, 2024).

Surprisingly, the amino acid sequence of the exon 4a protein shows very low conservation, even among mammals (e.g., only ∼50% sequence identity between primates and rodents), and almost no sequence identity with non-mammalian vertebrates (birds, reptiles, amphibians, fish). Nevertheless, the size of the 4a insert remains nearly invariant (e.g., 252 residues in humans and 257 in zebra finch). This contrasts sharply with the remainder of the tau protein, where both the N- and C-termini are highly conserved and the microtubule-binding domain (MTBD; encoded by exons 9–12) maintains strong sequence identity across vertebrate species (e.g., ∼95% between human and zebra finch) (Fischer, 2022). A larger form of the 4a exon termed 4a-L of was found in prostate cancer cell lines (Souter and Lee, 2010).

As a follow up we decided to investigate the structural properties of Big tau relative to LMW tau (human), and to better understand the unusual size conservation of a protein domain with little sequence homology, we analyzed and compared the two proteins with respect to charge distribution, hydrophobicity and aggregation propensity using a variety of computational tools (Housmans et al., 2023). Our goals were: (1) to determine the effect of exon 4a on tau structure (e.g., folding and aggregation) and (2) to assess these properties in an evolutionary context.

Our analyses revealed that exon 4a protein is characterized by a higher overall negative charge than the rest of tau (which contains a greater proportion of positive charges) and is more hydrophilic than LMW tau (which is overall more hydrophobic). These properties suggest that, while LMW tau is prone to aggregation through misfolding and aggregation mediated hydrophobic interactions, the exon 4a protein is relatively resistant to such aggregation. Importantly, incorporation of exon 4a into LMW tau to form Big tau shifts the hydrophobic properties of LMW tau toward greater hydrophilicity, with values intermediate between LMW tau and exon 4a protein. Analysis of the secondary structure verifies the potential effects of inclusion of 4a exon on aggregation, showing decrease in β strands. This likely reflects the strong influence of the ∼250 amino acid exon 4a insertion on the overall structural profile of Big tau. Consequently, we posit, the misfolding and aggregation potential of Big tau appears to be reduced even under pathological condition of mutations and hyperphosphorylation.

Another key finding is that the negative charge properties of exon 4a are conserved across species, while the hydrophilic properties vary. Despite low sequence conservation, the size and the structural feature of negative net charge is consistently found in different vertebrate species from amphibians to mammals, which often maintain a hydrophilic structure, suggesting evolutionary pressure to maintain these properties that ultimately define Big tau.

## Materials and methods

1. **Protein sequences:** Human tau isoforms and exon 4a sequences were retrieved from UniProt and Ensembl as previously described (Fischer, 2022). Comparative sequences were obtained for rat, zebra finch, and frog as follows:

***Homo sapiens*** microtubule-associated protein tau (MAPT)

ENST00000415613.6 MAPT-205, UniPort:P10636-9

**Big tau**

**Exon structure**

1. MAEPRQEFEVMEDHAGTYGLGDRKDQGGYTMHQDQE GDTDAGLK
2. ESPLQTPTEDGSEEPGSETSDAKSTPTAE
3. DVTAPLVDEGAPGKQAAAQPHTEIPEGTT
4. AEEAGIGDTPSLEDEAAGHVTQ

4a. EPESGKVVQEGFLREPGPPGLSHQLMSGMPGAPLLPE GPREATRQPSGTGPEDTEGGRHAPELLKHQLLGDLHQEGPPL KGAGGKERPGSKEEVDEDRDVDESSPQDSPPSKASPAQDGRPP QTAAREATSIPGFPAEGAIPLPVDFLSKVSTEIPASEPDGPSVGR AKGQDAPLEFTFHVEITPNVQKEQAHSEEHLGRAAFPGAPGE GPEARGPSLGEDTKEADLPEPSEKQPAAAPRGKPVSRVPQLK

5. ARMVSKSKDGTGSDDKKAK
6. TSTRSSAKTLKNRPCLSPKHPTPGSSDPLIQPSSPAVCPEPP SSPKYVSSVTSRTGSSGAKEMKLK
7. GADGKTKIATPRGAAPPGQKGQANATRIPAKTPPAPKTP PSS
8. ATKQVQRRPPPAGPRSER
9. GEPPKSGDRSGYSSPGSPGTPGSRSRTPSLPTPPTREPKKV AVVRTPPKSPSSAKSRLQTAPVPMPDLKNVKSKIGSTENL KHQPGGGK
10. VQIINKKLDLSNVQSKCGSKDNIKHVPGGGS
11. VQIVYKPVDLSKVTSKCGSLGNIHHKP
12. GGGQVEVKSEKLDFKDRVQSKIGSLDNITHVPGGGNKK
13. IETHKLTFRENAKAKTDHGAEIVYKSPVVSGDTSPRHLSN VSSTGSIDMVDSPQLATLADEVSASLAKQGL

**Domain structure** (Supplementary Figure 1).

**4a exon**

EPESGKVVQEGFLREPGPPGLSHQLMSGMPGAPLLPEGPR EATRQPSGTGPEDTEGGRHAPELLKHQLLGDLHQEGPPLKGA GGKERPGSKEEVDEDRDVDESSPQDSPPSKASPAQDGRPPQTA AREATSIPGFPAEGAIPLPVDFLSKVSTEIPASEPDGPSVGRAKG QDAPLEFTFHVEITPNVQKEQAHSEEHLGRAAFPGAPGEGPE ARGPSLGEDTKEADLPEPSEKQPAAAPRGKPVSRVPQLK

(251)

4a-L

EELRVPGRQRKAPERPLANEISAHVQPGPCGEASGVSGPCL GEKEPEAPVLLTASLPQHRPVCPAPPP

TGGPQEPSLEWRQKGGDWAEKGPAFPKPATTAYLHTEPES GKVVQEGFLREPGPPGLSHQLMSGMPGAPLLPEGPREATRQPS GTGPEDTEGGRHAPELLKHQLLGDLHQEGPPLKGAGGKERPG SKEEVDEDRDVDESSLQDSPPSKASPAQDGRPPQTAAREATSIP GFPAEGAIPLPVDFLSKVSTEIPASEPDGPSAGRAKGQDAHLEFT FHVEITPNVQKEQAHSEEHLGRAAFPGAPGEGPEARGPSLGED TKEADLPEPSEKQPAAAPRGKPVSRVPQK (354)

**Rat** *Rattus norvegicus*

ENSRNOT00000042984.6, MAPT-207, UniProt: F1LST4

EPQKVEIFSQSLLVEPGRREGQAPDSGISDWTHQQVPSMSG APLPPQGLREATHQPLGTRPEDVERSHPASELLWQESPQKEAW GKDRLGSEEEVDEDITMDESSQESPPSQASLAPGTATPQARSVS ASGVSGETTSIPGFPAEGSIPLPADFFSKVSAETQASPPEGPGTGP SEEGHEAAPEFTFHVEIKASAPKEQDLEGATVVGAPAEEQKAR GPSVGKGTKEASLLEPTDKQPAAGLPGRPVSRVPQLK

(254)

**Zebra Finch**

ENSTGUT00000021320.1 MAPT-208

GEPSSPKLQPGPRERVGEAVKSASQPPEQGLGPQQPPLSRET KAPAAAPTRIEVTIPIPLDMYQGSEGSGELWDQGGTEGLARAG GTGGHKDGPSPLCARATIKEDSGGRERDEDRDIDETSGQGLPSL VDQCVSLAPEGSCPAAAQEAREEYDGENKSKGVLRDTPGEALL VEAESHKAGEDQEEKRELLEGEGGPDSALSEPSGSVSLKEAEPRE GEDSGPVLETAKLPAEGEDGVKKVDEDAPVGEAVPDAGGRRTP RRKPGGLAADKASRVPLLK

(279)

**Frog** tropical clawed frog, *Xenopus tropicalis*

ENSXETT00000084149.2 MAPT-206, UniProt: A0A6I8RGV8

EEIALLAAAGQEEEYEMDTMEETLKITAKDQTHAENYGIT GDVDGESQNDETALSSGMVESAVEEDYYKETNGKEVNLEICE DDTEGWEEQIDEGIIMQDSVAPPKGGEQELSSVEQPQTNGTG AEHIFLEDNQHKKDTEEPFMAIPANSFPVGQIRPRASVSVYQV EIDANIPIDSKEAPCEDVGIPGGTKVDTERATEETLKSPRKRMP AHGSGIPVSRVPVPK

(273)

1. **Charge distribution:** Acidic and basic residues were quantified, and cumulative charge profiles were calculated and plotted https://www.bioinformatics.nl/emboss-explorer. Ratios of acidic to basic residues were calculated per domain.
2. **Hydrophobicity analysis:** The Kyte-Doolittle plot is a simple but powerful method to visualize hydrophobic vs. hydrophilic regions of a protein sequence, providing insights into folding, solubility, membrane-association, and aggregation (Kyte and Doolittle, 1982). We used the typical sliding window of 9 amino acids. After applying it to LMW tau, exon 4a protein, and Big tau, average hydrophobicity values were calculated followed by the plotting and analysis of the different domains
3. **Aggregation propensity:** Secondary structure and β-sheet formation were predicted with PASTA 2.0. Amyloid-prone regions were identified with AggreProt and Aggrescan. PASTA 2 (Walsh et al., 2014) is an energetic function derived from high-resolution protein structures, which considers interaction potential and H-bond formation between all non-consecutive residues for parallel and anti-parallel β -pairing (Walsh et al., 2014).
4. **AggreProt** (Planas-Iglesias et al., 2024) a machine learning sequence-based predictor of Amyloid-Prone Regions (APRs), was designed to detect short, amyloid-related and biologically relevant APRs, no longer than 50 residues (Planas-Iglesias et al., 2024). Aggrescan prediction (Kuriata et al., 2019) is assayed against an aggregation propensity scale for the 20 proteinogenic amino acids derived from *in vivo* experiments (Conchillo-Sole et al., 2007).
5. **Tango** was used to confirm the biophysical properties of the exon 4a protein, specifically its charge and hydrophilicity - http://tango.crg.es/,
6. **Evolutionary analysis:** Pairwise sequence identity was determined with Clustal Omega https://www.ebi.ac.uk/jdispatcher/msa/clustalo. Charge and hydrophobicity profiles were compared across species.
7. **Jalview** was used to display the alignments (Procter et al., 2021).
8. **NetPhos 3.1** (Blom et al., 1999) was used to predict phosphorylation sites and set to a high threshold to achieve agreement with other predictive programs.
9. **AlphFold2** predicts 3D protein structures from amino acid sequences using deep learning and evolutionary information (Jumper et al., 2021).

## Results

### Comparing LMW tau and exon 4a (human)

#### Charge distribution

**FIGURE 1:**
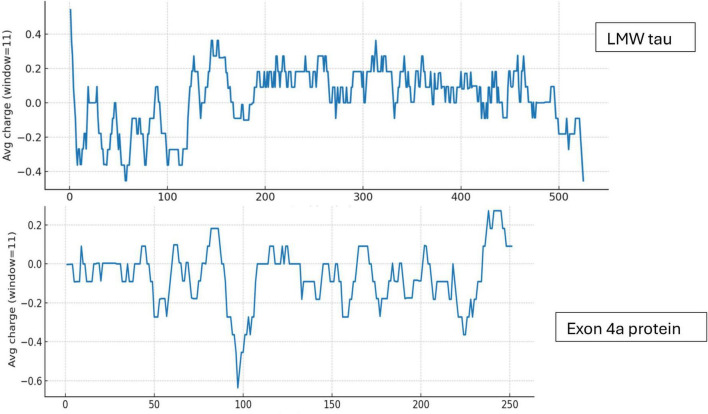
Charge distribution of LMW tau and exon 4a protein.

We examined the charge distribution, which is a critical determinant of protein structure, function, and molecular interactions. As shown in Figure 1, LMW tau contains a relatively balanced number of acidic and basic residues, with a slight excess of negative residues and charge distribution varying across domains. In contrast, exon 4a protein shows a significantly higher number of acidic residues compared to basic ones, resulting in a strong overall negative charge. The ratio of negative to positive residues underscores this difference: LMW tau has a ratio = 1.11, exon 4a 1.3 and Big tau has a ratio of 1.18 about midway between LMW tau and exon 4a as shown in Table 1. Further analysis related to electrostatic properties (Castro et al., 2019) are shown in Supplementary Figure 2 showing the estimated pI = 6.64 and a net charge at pH 7.0 = −2.25.

**TABLE 1:** Analysis of amino acids charges (negative/positive).

Protein	Length	# Negative	# Positive	Ratio negative/ positive
Exon 4a	251	43	33	1.30
LMW tau	525	61	55	1.11
Big tau	776	103	87	1.18

#### Hydrophobicity Profile

**FIGURE 2:**
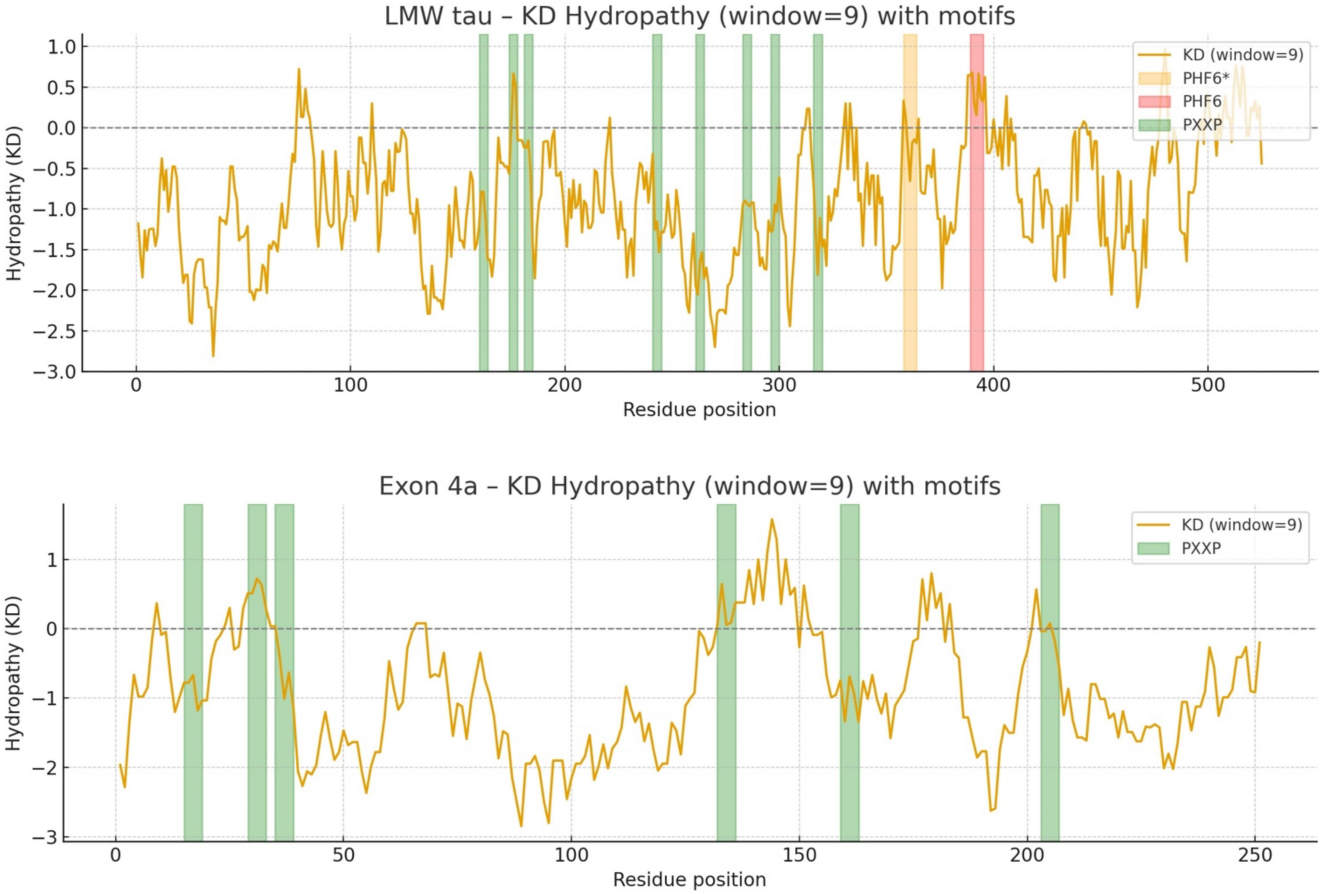
The Kyte-Doolittle hydrophobicity plot annotated with PHF6* and PHF6 as key aggregation “hot spots” and PXXP motifs as potential binding partners and regulatory interactions.

The distribution of hydrophobic versus hydrophilic residues strongly influences protein folding and structural stability. Using the Kyte-Doolittle scale, Figure 2 illustrates average hydrophobicity values. Scores closer to zero or positive reflect hydrophobicity, while more negative scores indicate hydrophilicity. As shown in Figure 2, LMW tau displays a mixed profile, with both hydrophobic and hydrophilic regions. Several peaks above the zero line reflect hydrophobic stretches that could form buried cores or, if sufficiently long, potential transmembrane segments. In contrast, the exon 4a protein remains consistently below zero, with only minimal hydrophobic peaks. This indicates a predominantly hydrophilic nature, consistent with its strong negative charge, suggesting that exon 4a protein is a soluble protein localized to aqueous environments or exposed surfaces of larger complexes. The average hydropathy value for LMW tau is −0.8930 while the exon 4a protein is −0.925. Note that the “hot spots” of aggregation in the LMW tau are shown in more details in Table 1.

#### Analysis of different LMW tau domains

**FIGURE 3:**
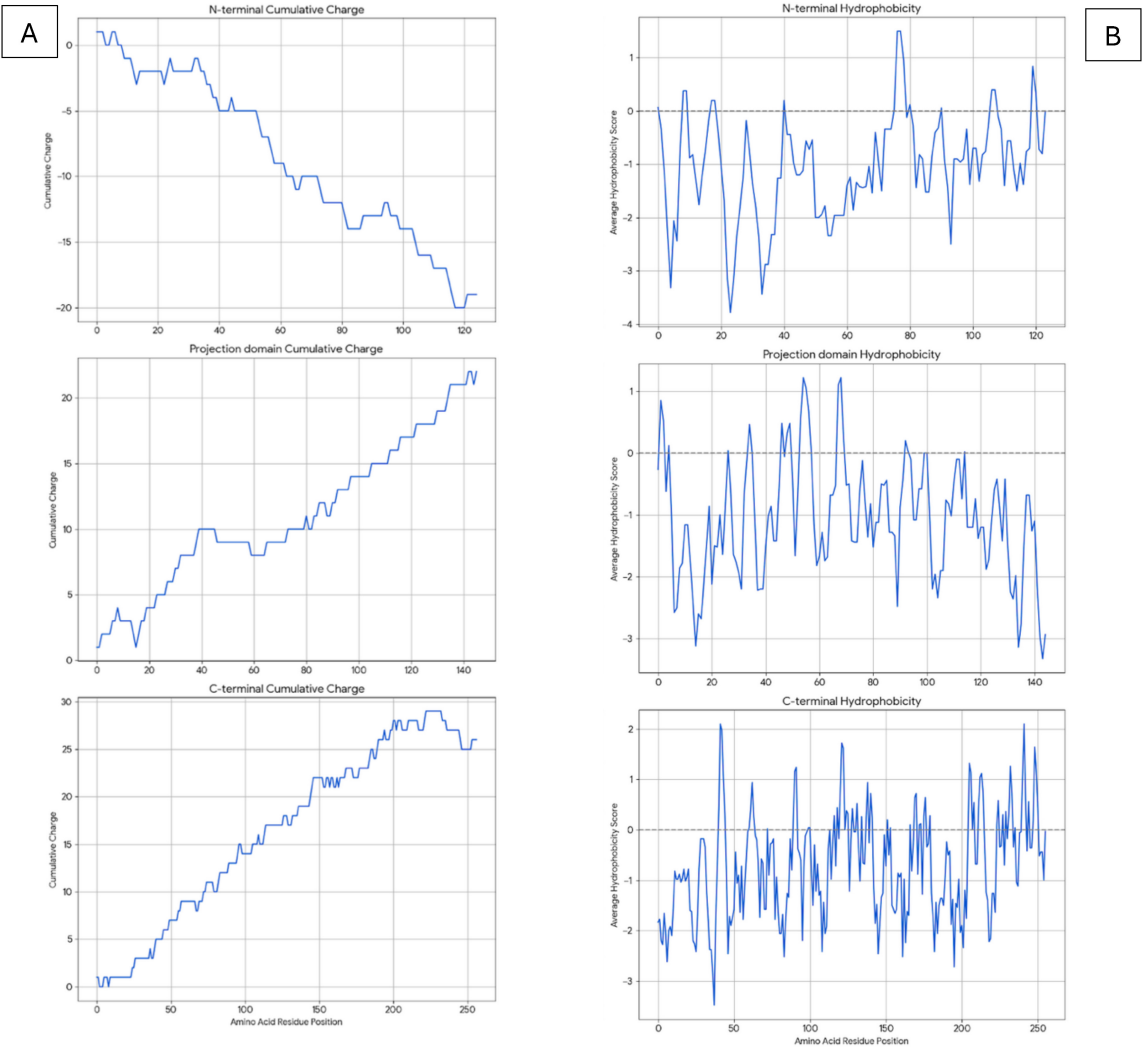
Analysis of LMW tau domains for charge distribution **(A)** and hydrophobicity **(B)** relative to amino acid position in *x*-axis.

To obtain more detailed information than the average values of LMW tau we analyzed the 3 major domains of tau as shown in Figure 3 for the ratio of negative to positives residues in Panel A and hydrophobicity values in Panel B.

**N-terminal**: The cumulative charge plot for the N-terminal protein shows a steep and consistent downward trend. This confirms its highly acidic nature, as negative charges accumulate rapidly along the sequence with an average ratio of 3. The hydrophobicity plot for this protein remains consistently in the negative range, indicating a highly hydrophilic and water-soluble peptide with a value of −1.0573. The fluctuations are minimal, which is typical for proteins that are largely disordered.

**Projection Domain:** The cumulative charge plot for the projection domain shows a steep and consistent upward trend. This reflects its highly basic nature, as positive charges accumulate along the sequence resulting in a low ratio of 0.28. Like the N-terminal peptide, this domain is also highly hydrophilic, with its hydrophobicity plot remaining consistently in the negative range at −1.0717. Its average hydrophobicity is the lowest of the three, making it the most hydrophilic overall.

**C-terminal**: The cumulative charge plot for the C-terminal protein shows a steep and consistent upward trend. This reflects its highly basic nature, as positive charges accumulate along the sequence resulting in a low value of 0.47. While still in the hydrophilic range, this protein is significantly less hydrophilic than the N-terminal protein with a value of −0.7121. The plot shows several distinct hydrophobic peaks that rise closer to the zero line, suggesting the presence of more hydrophobic regions including the aggregation “hot spots” of PHF6* and PHF6 shown in Figure 2 and Table 1.

#### Analysis of LMW tau aggregation

The aggregation propensity of LMW tau was examined using the AggreProt program which selected four different domains shown in Table 2. It highlights the known high aggregation domain of the MTBD repeats, which include the PHF6 motifs (Ganguly et al., 2015).

**TABLE 2:** Aggregation analysis with sequence features for the different domains of LMW tau.

Region	Sequence features	Aggregation tendency
N-terminal (1–150)	Acidic, Pro/Gly-rich	Low, mostly disordered, soluble
Projection domain (151–244)	Many PXXP motifs, positive charges	Low-moderate, can regulate conformational transitions
MTBD (245–368)	Contains PHF6* and PHF6	Very high, nucleation core
C-terminal (369–441)	Acidic, charged, extended	Moderate-low, stabilizes but not strongly amyloidogenic

The PXXP motifs can be protein–protein interaction domains with SH3 containing partners, role in signaling and cytoskeletal regulation. The various PHF6 domains drive abnormal aggregation into paired helical filaments (PHFs) hallmark of Alzheimer’s disease and other tauopathies.

### Comparing Hydrophobicity of LMW tau and Big tau

#### Hydrophobicity

**FIGURE 4:**
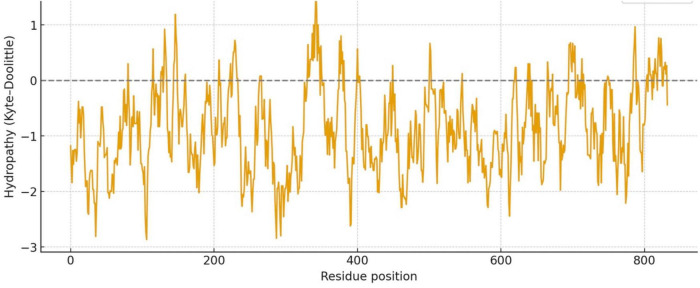
Hydrophobicity plot of Big tau (LMW tau + exon 4a).

Given the marked structural differences between LMW tau and exon 4a, we next examined Big tau, which incorporates the exon 4a sequence. Hydrophobicity analysis revealed that Big tau shows intermediate hydrophilic values between LMW tau and exon 4a, reflecting the substantial influence of the exon 4a domain. Thus, with the value of LMW tau at −0.8930 and exon 4a at −0.9259 the combined hydrophobicity value of Big tau showed at −0.9036 which is the average value between LMW tau and exon 4a: −0.9094. On the Kyte–Doolittle scale (Figure 4), this shift is most evident between residues 120–375, corresponding to the exon 4a region. These values confirm that inclusion of exon 4a alters the global hydrophobicity of tau, reducing the overall hydrophobic properties of tau.

#### Analysis of aggregation propensity

**FIGURE 5:**
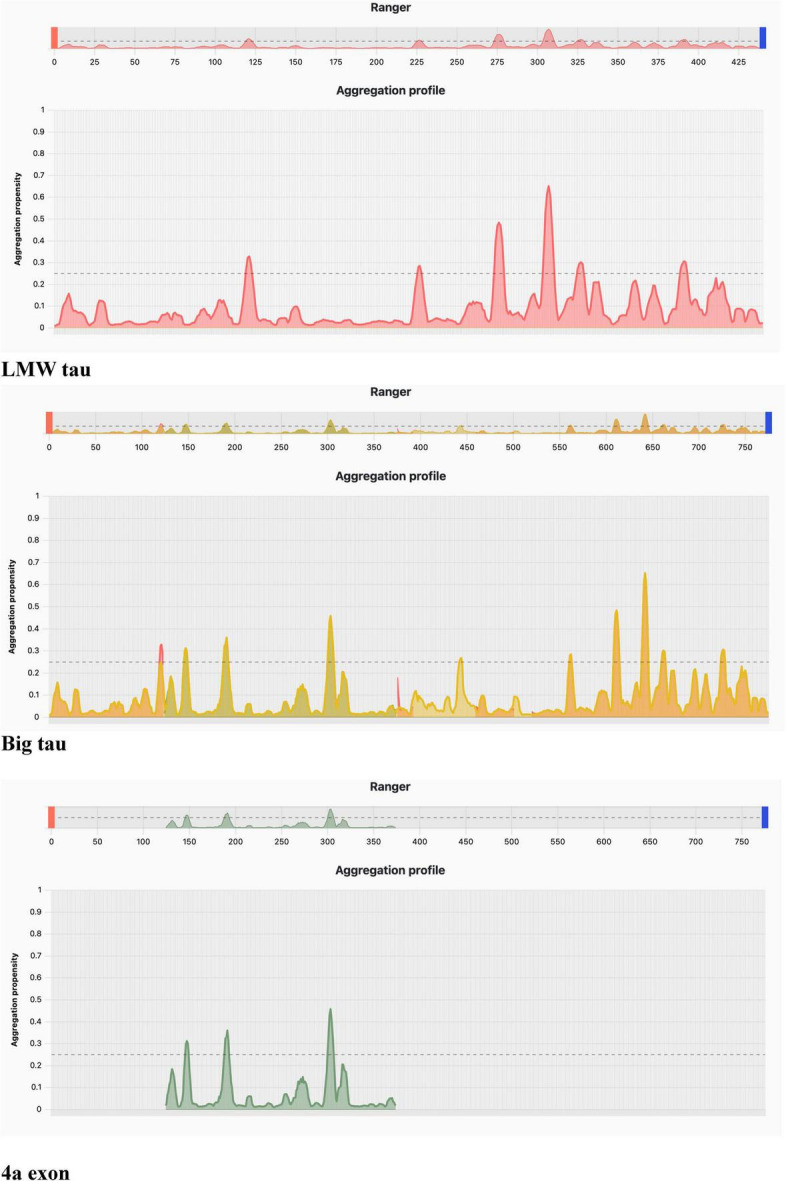
Aggregation profile using AggreProt.

Using PASTA 2 analysis we determined the secondary structure of the protein with focus on β-sheet–forming sequences are the primary drivers of aggregation (Table 3). Once formed, β-sheets template further misfolding, creating a self-propagating process. Mutations that increase β-sheet propensity (e.g., replacing polar residues with hydrophobic ones) usually enhance aggregation. Exon 4a showed a much the lower values for β-sheet relative to LWM tau (4.78 vs. 17.33), which resulted in Big tau a lower value, a decrease of almost 25% (13.14 vs. 17.33). Interestingly, the exon 4a-L had a higher value of β-sheet relative to 4a suggesting that it may not be as effective in reducing the aggregation potential. The aggregation propensity is also shown in Figure 5 with a plot generated by AggreePlot for LMW tau, exon 4a and Big tau.

**TABLE 3:** Secondary structure analysis PASTA 2 with emphasis on % β-strand.

Protein	Length	# Amyloids	Best energy	% Disorder	% α -helix	% β -strand	% Coil
Exon 4a	251	0	−4.10	96.01	11.95	4.78	83.27
LMW tau	525	1	−5.51	68.19	6.86	17.33	75.81
Big tau	776	1	−5.51	78.47	8.25	13.14	78.61
Exon 4a-L	355	0	−4.10	91.54	7.89	6.2	85.92

#### Aggregation analysis using AggreProt

The analysis shows sharp peaks in the microtubule-binding repeat region underscoring that LMW tau has exposed aggregation-prone motifs. It also shows that the long N-terminal extension (exon 4a) contributes to a large aggregation-resistant region with a protective effect.

#### The 3D analysis of Big tau structure

**FIGURE 6:**
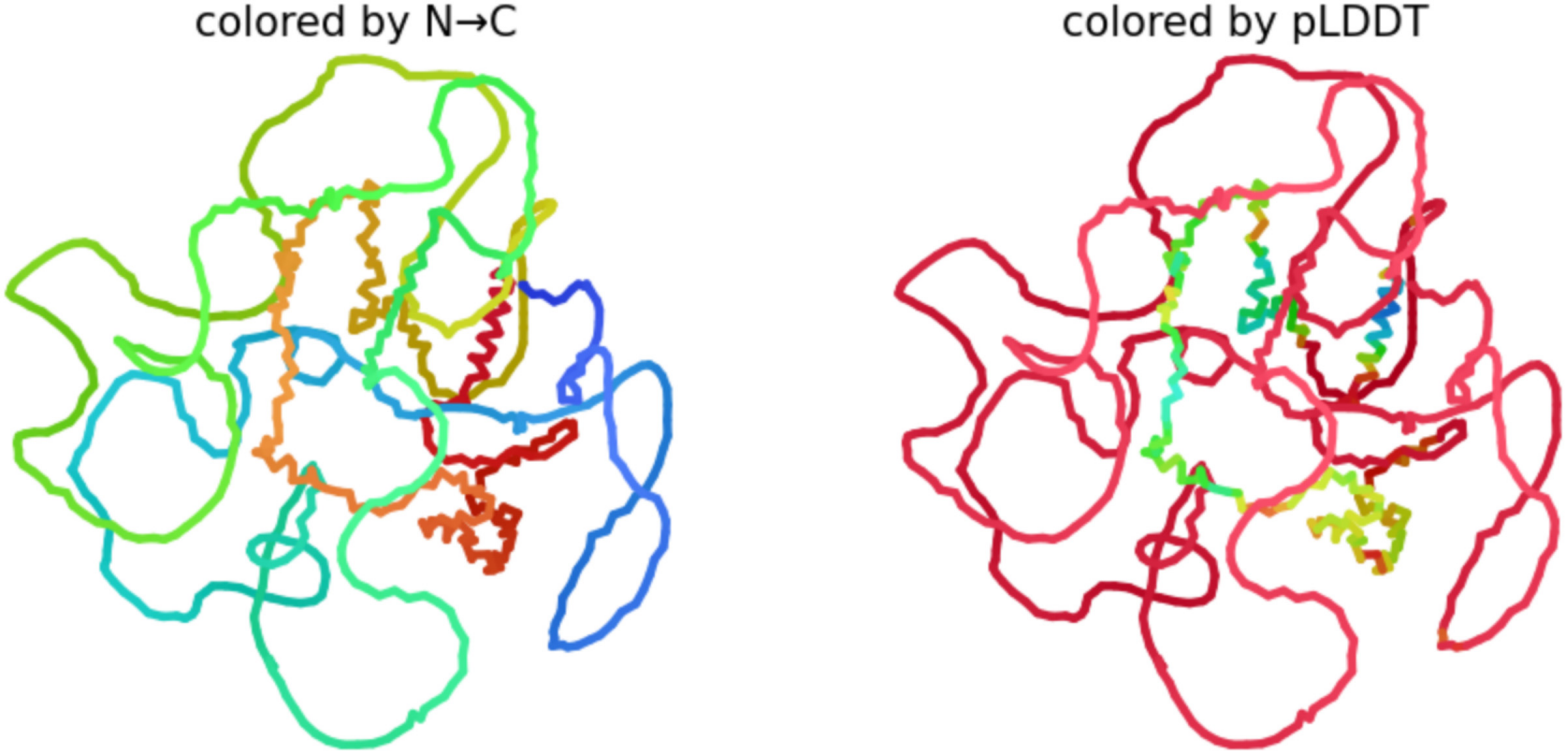
Predictive 3D structure of Big tau: colored by N-C uses the rainbow (blue → green → yellow → red) to represent the sequence order from the N-terminus to C-terminus.

We used AlphFold2 program to derive the predictive 3D model of Big tau as shown in Figure 6. The 3D analysis confirms that Big Tau is predicted to be disordered. The microtubule-binding repeat domain (C-terminal half) show slightly higher confidence (greenish regions) because it contains short β-strands. The N-terminal projection domain together with exon 4a insert are red and unstructured, supporting their role as flexible, extended spacers that could shield the regions of high aggregation propensity.

Because Big Tau has long disordered linkers (especially in the N-terminal and 4a insert regions), the structure looks loosely packed, with many loops. The Compact regions (e.g., small helices or turns) appear as short, tangled segments; the rest is extended and flexible. pLDDT (predicted Local Distance Difference Test score), shows the confidence AlphaFold assigns to each residue (range 0–100). Blue (90–100) likely a well-defined secondary structure (helix, β-strand), Green/yellow (50–80) moderate confidence; probably dynamic or partially structured, Red (<50) very low confidence; almost certainly intrinsically disordered.

#### Analysis of phosphorylation sites

**FIGURE 7:**
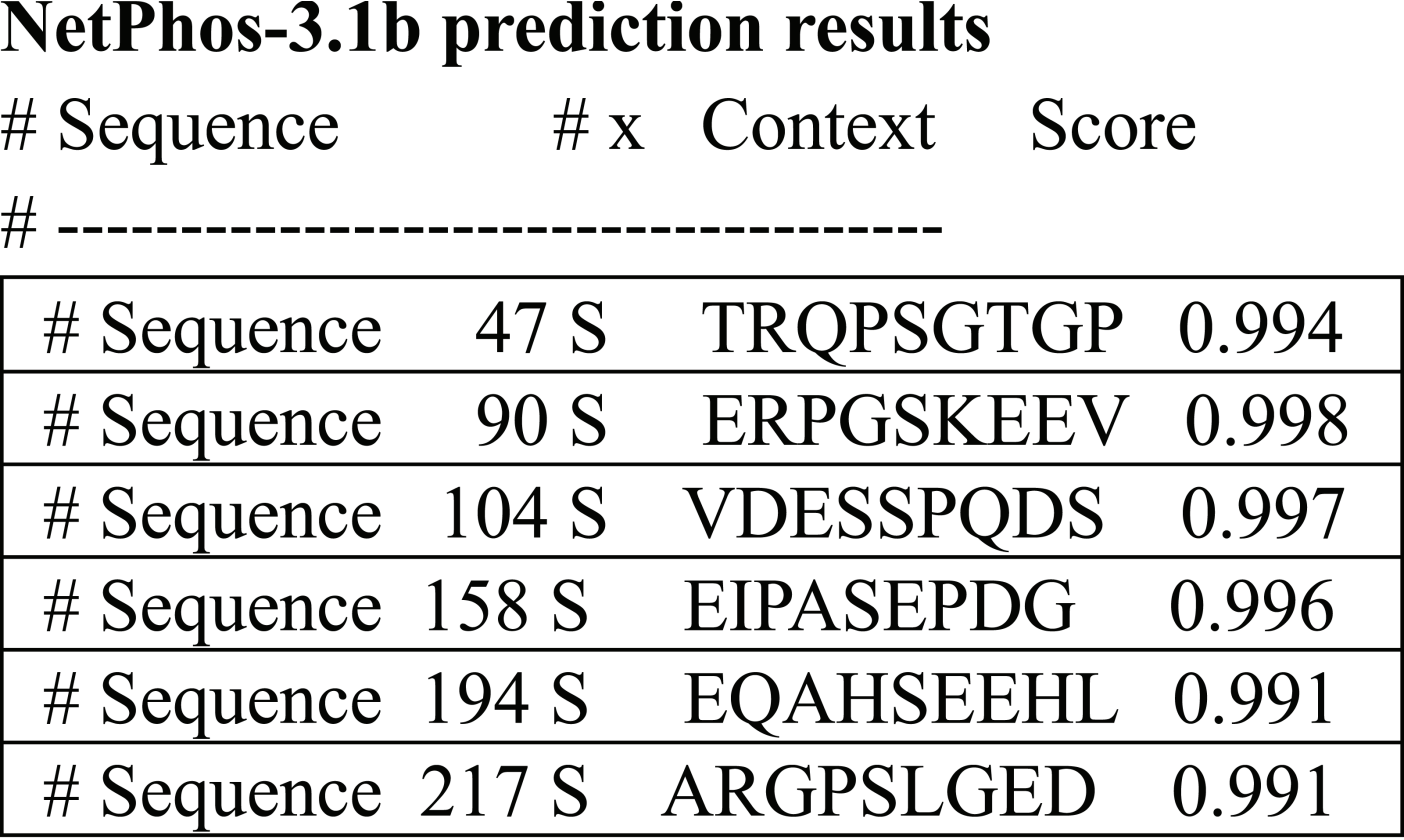
Prediction of high confidence phosphorylation sites using the 251aa sequence shown in see section “Materials and methods.”

We analyzed the prediction of high confidence phosphorylation regions on the 4a-derived protein and discovered 5 potential serine sites along the 251 amino acids of the human sequence (Figure 7). This is a much lower density than LMW tau composed of about 440 amino acids, known to be one of the highest phosphorylated protein with about 80 potential phosphorylation sites and over 40 experimentally verifies, some associated with tauopathies driven by hyperphosphorylation (Grundke-Iqbal et al., 1986; Trushina et al., 2019; Wegmann et al., 2021). Interestingly, despite low sequence conservation a similar low density was found in the 4a derived protein of other species (e.g., rat, zebra finch and frog) implying that these properties may have also been conserved.

### Evolutionary perspective

Previous studies have shown that the amino acid sequence of the exon 4a protein shows very low conservation, even among mammals (e.g., only ∼50% sequence identity between primates and rodents), and decreasing sequence identity with non-mammalian vertebrates <25% (birds, reptiles, amphibians, fish) confirmed in sequence alignments shown in Supplementary Figure 3. Nevertheless, the size of the 4a protein remains similar (ranging in the examples below 226–280). This contrasts sharply with the remainder of the tau protein, where both the N- and C-termini containing the MTBD maintains strong sequence identity across vertebrate species as shown in Table 4 and (Fischer, 2022).

**TABLE 4:** Analysis of sequence identity across vertebrates.

Sequence	Human	Rat	Zebra finch	Frog
LMW tau	100	71	52	43
N-terminal	100	83	60	38
C-terminal	100	97	91	80
4 exon protein	100	56	24	16

To assess whether the unique properties of human exon 4a protein are conserved across vertebrates, we analyzed charge ratio and hydrophobicity value of exon 4a protein in representative species:

**Exon 4a negative to positive ratio**

- Human: 1.3
- Rat: 1.58
- Zebra Finch: 1.56
- Frog: 2.32

**Exon 4a hydropathy values**

- Human: −0.9259
- Rat: −0.7677
- Zebra Finch: −0.9061
- Frog: −0.7770

All these exon 4a proteins displayed negative scores, confirming hydrophilicity as a conserved feature. Proteins with less negative values (rat and frog) exhibited more peaks approaching the zero line, reflecting localized hydrophobic stretches. Taken together, these results indicate that, despite low sequence conservation, exon 4a protein maintains consistent structural properties across vertebrate evolution: stable size, negative net charge, and hydrophilicity. These conserved features likely serve to mitigate tau misfolding and aggregation. A more detailed analysis of the different vertebrate classes is shown in Supplementary Table 1.

## Discussion

This study describes structural and evolutionary analyses of tau’s exon 4a, the defining feature of the high molecular weight Big tau protein (Fischer and Baas, 2020). Our findings address the longstanding question of how a protein domain with minimal sequence conservation can nonetheless maintain a consistent length and functional role across diverse vertebrate lineages (Fischer, 2022). Using computational tools which are sequence-based and structure-based such charge distribution, Kyte-Doolittle plot, PASTA 2.0, AggreProt Aggrescan and Tango (Navarro and Ventura, 2022; Housmans et al., 2023), we have shown that although the primary amino acid sequence encoded by exon 4a is highly divergent, its size of about 250 amino acids and key biophysical properties including charge distribution, hydrophobicity, and aggregation profile are conserved. These features counterbalance the inherent aggregation propensity of LMW tau, suggesting that exon 4a represents an evolutionary innovation to mitigate tau misfolding. Specifically, our analyses indicate that inclusion of exon 4a modifies the structural properties of tau by introducing a highly acidic, hydrophilic, and intrinsically disordered domain. Given that protein misfolding and aggregation are frequently driven by exposed hydrophobic patches, the insertion of such a hydrophilic, negatively charged domain likely reduces intermolecular association by increasing solvation and electrostatic repulsion (Levine et al., 2015; Hernandez et al., 2022). This also effectively reduces the β-sheet secondary structure and aggregation potential. We propose a model in which the large exon 4a-derived stretch of about 250 amino acids increases dramatically the projection domain to more effectively shield the PHF motifs and limiting their solvent exposure which together with its unique biophysical properties distinguishes the Big tau protein from the LMW tau. Importantly, the 4a exon is adjacent to the N-terminal which as shown in Table 2 is also an acidic, Pro/Gly-rich region mostly disordered and soluble with low aggregation propensity. Together with 4a they span 400 amino acids amplifying the proposed properties Big tau in counterbalancing the aggregation-prone domains of LMW tau even under pathological conditions of harmful mutations and hyperphosphorylation. These modifications likely affect the physiological roles of Big tau as well as attenuate pathological misfolding in regions of the nervous system with high expression of Big tau. One of these regions is the ventral spinal cord where Big tau is expressed in lower motor neurons (but not the upper motor neurons or upper spinal neurons), suggesting the potential as a specific biomarker.

Interestingly, exon 4a-L may not be as effective as 4a: Its average hydrophobicity is −0.8794 vs. −0.9259), which is similar to LMW tau, and it has a higher value of β-sheet than 4a suggesting that it may not be as effective in reducing the aggregation potential and thus play a different role possibly related to its larger size of 354 amino acids and less hydrophilic N-terminal domain. Indeed, 4a-L was identified in searches of MAPT orthologs mostly in primates and a few mammals (Fischer, 2022) but found experimentally only in prostate cancer cell lines where microtubules are mostly associated with cell division (Souter and Lee, 2010).

### Functional implications

The proposed model aligns with observations that Big tau is selectively expressed in neurons of the peripheral and autonomic nervous systems, as well as in specific CNS regions such as brainstem and cerebellum, which are typically less vulnerable to tauopathies (Boyne et al., 1995; Fischer and Baas, 2020; Chung et al., 2024). The biophysical properties of exon 4a protein may therefore represent a molecular adaptation to enhance proteostatic resilience in these neuronal populations, which must maintain long axons and high transport demands over the lifespan. This model also aligns with emerging therapeutic strategies aimed at modulating tau aggregation and interfering with seed propagation. Beyond its role in aggregation suppression which is the focus of this study, Big tau is may have different physiological properties than LMW tau with different impact on microtubule dynamics, axonal transport, and interactions with cytoskeletal proteins, which we have previously suggested but needs experimental evidence (Fischer, 2022). The expanded projection domain could increase inter-microtubule spacing, reduce crosslinking density, and alter bundling properties, thereby creating a cytoskeletal architecture optimized for long-range axonal viability. Moreover, by decreasing the density of tau molecules bound to microtubules, Big tau may reduce steric hindrance for motor proteins, generating a more permissive substrate for kinesin- and dynein-mediated transport.

There are also some structural motifs in the human 4a sequence that can interact with several specific proteins, primarily those involved in cellular signaling and cytoskeleton regulation. For example, the PXXP motifs have a potential for binding partners and regulatory interactions and the S/T-P motifs in the 4a sequence are targeted by specific kinases that phosphorylate these sites. These include CDK5, and MAPKs. The proline-rich motifs in the 4a sequence are binding sites for proteins containing SH3 such as Fyn kinase mediating its function at the synapse. Once phosphorylated, specific sites on the exon 4a sequence can be recognized by 14-3-3, which are adapter proteins that bind to a phospho-serine/threonine motif and regulate the activity, localization, or stability of their binding partner. However, as shown in Figure 6, the phosphorylation density of 4a protein is low and the effects of such putative post-translation modification may be limited (e.g., within mammals) because of low sequence conservation across vertebrates. The large 4a insert is therefore unlikely to contribute to the hyper-phosphorylation associated with the pathological state of tau. In fact, no phosphorylation at the 4a site has been experimentally verified. Searches of other motifs using ELM (The Eukaryotic Linear resource for functional sites in protein) confirmed the phosphorylation sites but in addition showed short often partial sequences related to protein degradation and ubiquitination, which we could not fully confirm.

### Evolutionary perspective

Comparative analyses of human, rat, zebra finch, and frog tau reveal a striking pattern where exon 4a shows extensive primary-sequence divergence yet contributes a highly conserved length and biophysical profile to the tau protein. This combination of low sequence identity with conserved composition is characteristic of IDR, which are often under selection for ensemble properties (e.g., charge density, hydrophobicity, proline/glycine content) rather than sequence motifs (Zarin et al., 2017; Holehouse and Kragelund, 2024; Singleton and Eisen, 2024). Our hydrophobicity analyses reinforce this view, showing that exon 4a protein is more hydrophilic than LMW tau, while Big tau as a whole occupies an intermediate state consistent with evolutionary pressure to preserve solubility and suppress β-sheet formation within the expanded projection domain. By contrast, the MTBD is highly conserved across species and contains short hexapeptides that drive aggregation (Ganguly et al., 2015). The exon 4a encoded region appears to act as a solubilizing, charge-rich spacer that offsets the aggregation risk conferred by the MTBD. This may be especially advantageous in peripheral nervous system neurons, which face extreme transport distances and high molecular crowding along axons.

The near-fixed length of exon 4a across birds, amphibians, and mammals suggests geometric or structural constraints: a shorter insertion might fail to provide sufficient solubilizing capacity, while a longer one could impose steric or metabolic costs. The convergent retention of charge density and hydrophilicity, despite sequence divergence, indicates that selection has operated on physicochemical properties rather than precise sequence motifs. This modular organization of conserved functional “core” domains (e.g., MTBD, C-terminal tail) combined with compositionally conserved IDR “spacers” underscores how tau balances structural versatility with proteostatic resilience. Although all the tested species maintain an overall hydrophilic, acidic profile in exon 4a protein, our analyses suggest that frogs and rats exhibit somewhat lower negative to subtle species-specific differences in chain compaction under physiological ionic conditions. Nonetheless, the preserved length and high overall charge imply that the fundamental role of exon 4a of aggregation suppression and structural spacing is conserved. Given the low sequence conservation of the 4a protein outside mammal species, these putative interactions may be limited to mammalian species in general and primates in particular reflected the high complexity of the nervous system.

From an evolutionary standpoint, exon 4a represents a proteostatic adaptation that buffers the aggregation-prone MTBD, particularly in neurons with long axons and high transport loads. This stabilizing, disorder-based module provides a blueprint for therapeutic design: acidic IDR-like extensions or charge-enhancing modifications grafted onto LMW tau could emulate Big tau’s resistance to misfolding without disrupting MT binding.

### Limitations and future directions

Our sequence-based and bioinformatic analyses provide a strong mechanistic framework for the proposal that the properties of Big tau are mediated by a conserved size of the exon 4a and the unique physicochemical structure of its coded protein, but experimental analysis will be essential for validation. For example, *in vitro* aggregation assays, cellular neuronal cultures, and *in vivo* animal models should be employed to test whether the expression of Big tau exhibits lower aggregation kinetics associated with functional resilience in vulnerable neurons even under pathological conditions affecting the CNS and explore its specific physiological function in peripheral neurons.

## Conclusion

Exon 4a defines the unique properties of Big tau by introducing a large, acidic, and hydrophilic domain that counterbalances the aggregation-prone domains of LMW tau. It shifts tau’s role from a more dynamic microtubule regulator (e.g., LMW tau in CNS) to a structural stabilizer in long projection peripheral axons requiring more stable, extended spacing between microtubules. Functionally, this explains why Big tau is less associated with pathological aggregation and might represent an evolutionary adaptation to axon length and stability. Despite extensive sequence divergence, the conservation of exon 4a length and charge distribution across vertebrates underscores its evolutionary significance. Despite low primary sequence conservation across vertebrates, exon 4a homologs retain a conserved architecture of a large, insert enriched in acidic residues that is predominantly disordered and hydrophilic. By conferring resistance to misfolding, Big tau may help explain the relative resilience of peripheral and cerebellar neurons to tau-related neurodegeneration.

## Data Availability

The datasets presented in this study can be found in online repositories. The names of the repository/repositories and accession number(s) can be found in the article/Supplementary material.
