# Supplementary material for: Analyses of exon 4a structure reveal the properties of Big tau related to distribution, function and aggregation
Source: Front Mol Neurosci. 2025 Oct 28;18:1707820. doi: 10.3389/fnmol.2025.1707820 (PMC12602400; doi:10.3389/fnmol.2025.1707820)
Supplement: Supplementary file 1 [file Data_Sheet_1.docx]

**Analyses of exon 4a structure reveal the properties of Big tau related to distribution, function and aggregation**

**SUPPLEMENTARY DATA**

**Supplemental Table 1: Comparative properties of 4a across vertebrate species**

| **Vertebrate Group** | **Length (aa)** | **Charge/ Disorder** | **Hydropathy** | **Aggregation Propensity** | **Identity to Human** | **Qualitative Notes** |
| --- | --- | --- | --- | --- | --- | --- |
| **Primates** Homan, Chimp | 252–257 | acidic, intrinsically disordered | Hydrophilic | Low aggregation | 95–100% | Nearly identical; conserved motifs; |
| **Other Mammals** Mouse, rat | 250–260 | Acidic, disordered | Hydrophilic | Low aggregation | 50–60% | Conserved charge; less sequence identity, similar biophysical profile. |
| **Birds** Zebra finch, Chick | 255–280 | Acidic, high disorder | Hydrophilic | Low aggregation | 25–30% | Divergent sequence, fewer conserved motifs; still low aggregation. |
| **Reptiles** Green anole, turtle | Variable 255–265 | Acidic, disordered | Generally hydrophilic but variable | Low - moderate | 20–30% | divergence; some loss motifs. |
| **Amphibians** Xenopus laevis, Xenopus tropicalis | 250–270 | Acidic, highly disordered | Hydrophilic with local hydrophobic peaks | Low - moderate | 15–25% | Substantial divergence; many substitutions; keeps net disorder. |
| **Teleost Fish** Zebrafish, Medaka | 260–320 | Strongly acidic, long disordered stretches | Variable; often less hydrophilic | Variable | 10–20% | Highly divergent; exon 4a - large acidic insert, motifs poorly conserved. |
| **Cartilaginous Fish.** Shark, Skate | Likely absent, highly diverged |  |  |  |  | Earliest divergence; mostly length- and charge-driven conservation, not sequence. |
| **Jawless Fish** Lamprey, Hagfish | Likely absent, no clear homolog |  |  |  |  | Very low homology to human MAPT |

**Legend:** Length = exon 4a-like insert. Charge = overall net acidic tendency (negative = acidic). Hydropathy = overall Kyte–Doolittle sign (Hydrophilic = negative avg). Disorder = predicted fraction of residues called disordered (high/moderate/low). Aggregation = short-aggregation-prone-regions (low/moderate/high).

Supplemental Fig. 1: Structural domains of Big tau

NTR-N-terminal, MTBD-microtubule binding domain, CTR-C-terminal

Supplemental Fig. 2


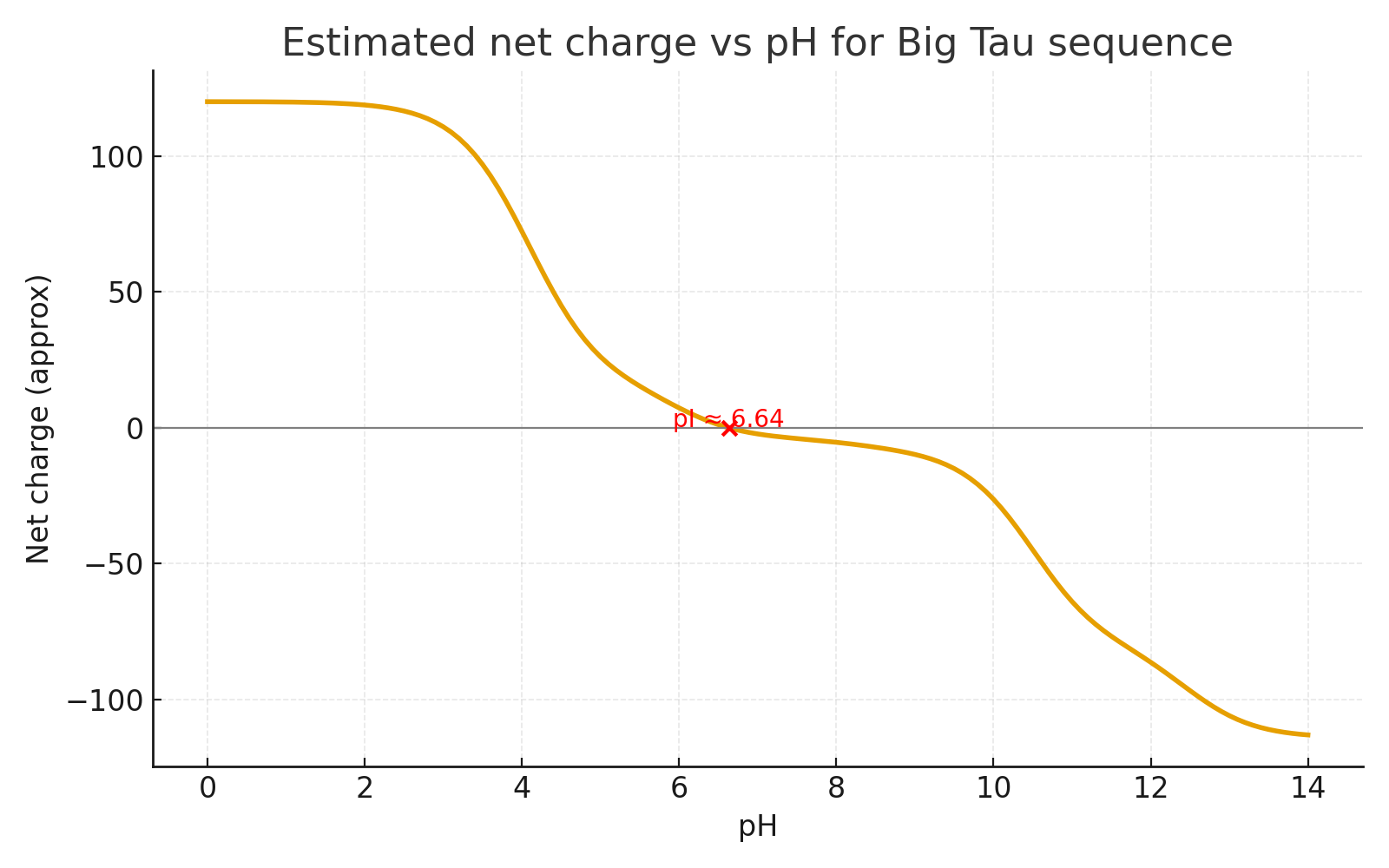


The estimated pI=6.64 (linear interpolation between points where net charge crosses zero) with a net charge at pH 7.0= −2.25 (slightly net-negative at physiological pH). This is a simplified, sequence-based plot not a 3D analysis that accounts local environment shifts of buried residues, or conformational ensembles.

Supplemental Fig. 3: alignment of 4a sequence against human #1by order for rat #2, zebra finch #3,and frog #4..


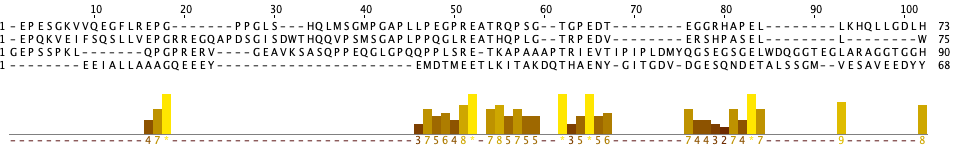


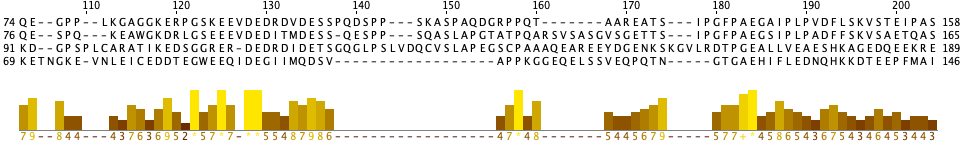


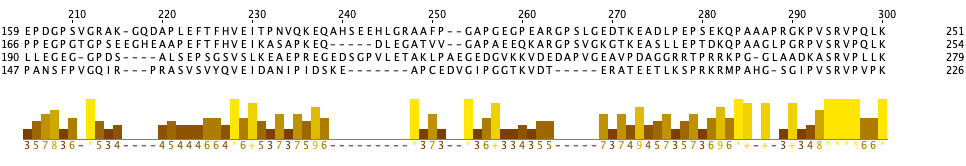


Legend: Yellow: conservation al 4 sequences, Beige 3, brown 2.

As previously discussed, despite low primary sequence conservation across vertebrates, exon 4a homologs retain a conserved architecture: a large, low complexity insert enriched in acidic residues that is predominantly disordered and hydrophilic suggesting convergent biophysical properties rather than strict sequence motifs. As shown in the supplemental table, in mammals and many tetrapod, exon 4a or analogs are present as a ~250–260 amino acid insert that markedly extends the projection domain. In many non-mammalian vertebrates, the exon may be present, but sequence diverged. The length tends to be conserved in mammals but primary sequence identity is low across deep phylogeny, explaining earlier observations of ~50% identity between primates and rodents, <25% with non-mammals (Fischer, 2022). Biophysical properties are also conserved (especially among primates) with a net negative charge as the most conserved feature across many vertebrates showing that exon 4a proteins are more acidic than the rest of tau. In addition, exon 4a is generally hydrophilic though the degree varies by species. Finally, exon 4a protein generally shows low aggregation propensity. Across vertebrates, exon 4a-like inserts appear to provide a soluble, acidic spacer that increases projection-domain length and likely reduces aggregation risk, a convergent functional solution even when sequence diverges. In teleost fishes and more so in cartilagous and jawless fishes the exon 4a orthologs show no homology to the mammalian counterpart, suggesting that this exon may have evolved independently in different species (Fischer, 2022).

Fischer, I. (2022). Evolutionary perspective of Big tau structure: 4a exon variants of MAPT. *Front Mol Neurosci* 15**,** 1019999.
